# Assessment of Serum Acylated Ghrelin in Children and Adolescents with Chronic Liver Diseases: Relation to Nutritional Status

**DOI:** 10.1155/2014/560516

**Published:** 2014-10-14

**Authors:** Z. A. Elkabbany, R. T. Hamza, N. H. Mahmoud

**Affiliations:** ^1^Departments of Pediatrics, Faculty of Medicine, Ain Shams University, Cairo, Egypt; ^2^Pediatrics and Pediatric Endocrinology, Faculty of Medicine, Ain Shams University, Cairo, Egypt; ^3^Clinical Pathology, Faculty of Medicine, Ain Shams University, Cairo, Egypt

## Abstract

Because ghrelin is one of the key hormones in regulating feeding behavior and caloric status, it was suggested that ghrelin behavior might be closely associated with malnutrition state of patients with chronic liver disease (CLD). Thus, we aimed to assess serum ghrelin levels in children with CLD and its relation to anthropometric parameters and severity of CLD. Forty CLD patients were studied in comparison to 40 controls. All subjects were subjected to history, anthropometric, and laboratory assessment of liver functions and serum acylated ghrelin. Ghrelin was higher in patients than controls being higher with progress of Child's grade and with deterioration of liver functions. Hyperghrelinemia was detected in 62.5% of cases. Ghrelin correlated negatively with body mass index standard deviation score (BMISDS (*r* = −0.95, *P* < 0.001)), triceps skin fold thickness (TSFT (*r* = −0.88, *P* < 0.001)), and subscapular skin fold thickness (SSFT (*r* = 0.83, *P* < 0.001)) percentiles. In conclusion, hyperghrelinemia may represent a compensatory mechanism trying to overcome malnutrition state complicating CLD and can be used as a parameter for early detection and assessment of the severity of malnutrition in children with CLD.

## 1. Introduction

CLDs are common pediatric problems in children in developing countries. They are characterized by numerous metabolic alterations, predominantly catabolic, resulting in the clinical picture of malnutrition or even cachexia and contributing to complications such as hepatic encephalopathy [1]. The mechanisms of malnutrition in CLD are not completely understood. Both poor dietary intake and increased basal energy expenditure have been reported to contribute to a negative energy balance in such patients [2]. In cirrhotic patients, liver failure causes both decreased protein synthesis and enhanced protein breakdown, which together with anorexia and reduced food intake can lead to severe protein energy malnutrition (PEM) and limit the capacity for regeneration and functional recovery of the liver [3].

Ghrelin is an endogenous ligand of the growth hormone secretagogue receptor (GHS-R) and a potent stimulator of growth hormone (GH) release in humans. It is synthesized primarily by the stomach and in substantially lower amounts by the bowel, pituitary, kidney, placenta, and hypothalamus. Ghrelin anticipates the initiation of meals and releases GH (growth hormone), and it has been postulated that ghrelin integrates anabolic changes in the body [4].

Because ghrelin is one of the key hormones in regulating feeding behavior and caloric status, it was suggested that ghrelin behavior might be closely associated with protein energy malnutrition (PEM) in patients with liver cirrhosis since plasma ghrelin levels reflect the malnutrition state of patients with CLD and could be related to anthropometric parameters in those patients [5]. In catabolic situations like in cirrhosis, raised ghrelin levels may induce a combination of enhanced food intake, increased gastric emptying, and food assimilation [3].

Thus, the current study aimed to assess serum ghrelin levels in children with CLD and its relation to anthropometric parameters and severity of CLD.

## 2. Subjects and Methods

### 2.1. Study Population

This cross sectional case-control study was conducted during the period from the beginning of September 2012 to the end of May 2013. It included 40 Egyptian patients with CLD, regularly attending the Pediatric Hepatology Clinic, Children's Hospital, Ain Shams University, Cairo, Egypt. They were 22 males and 18 females with a mean age of 7.55 ± 3.46 years (range: 1–16 years).

Patients were classified according to Child-Turcotte-Pugh classification [6] which uses clinical and laboratory information to stratify disease severity, surgical risk, and overall prognosis. Risk (grade) is based on the total number of points scored and is classified into low (A): 5-6, moderate (B): 7–9, and high (C): 10–15 points. In group A, there is no encephalopathy or ascites, bilirubin < 1.5 mg%, albumin > 3.5 g/dL, and prothrombin time was 10–14 seconds; group B had mild to moderate encephalopathy, slight ascites, bilirubin 1.5–3 mg%, albumin 2.8–3.5 g/dL, and prothrombin time 14–16 seconds, and group C had encephalopathy, moderate ascites, bilirubin > 3 mg%, albumin < 2.8 g/dL, and prothrombin time >16 seconds.

Group A included 15 patients (9 males and 6 females) with a mean age of 6.6 ± 4.5 years (range: 1–14 years), group B included 15 patients (8 males and 7 females) with a mean age of 5.9 ± 6.2 years (range: 0.5–15 years), and group C included 10 patients (5 males and 5 females) with a mean age of 7.2 ± 3.0 years (range: 1–16 years).

Patients were compared to 40 age-, sex- and pubertal stage-matched apparently healthy children (22 males and 18 females) whose mean age was 7.6 ± 2.5 years ranging between 1 and 15.5 years.

Children with malignancies, bacterial infections, chronic malabsorption, impaired kidney functions, endocrinal problems, or any other chronic systemic illness that could affect growth or nutritional status were excluded from the study.

An informed written consent was signed by the parents or legal guardians of the studied subjects. This study was approved by the Ethics Committee, Faculty of Medicine, Ain Shams University, Cairo, Egypt, which complies with the World Medical Association Declaration of Helsinki regarding ethical conduct of research involving human subjects and/or animals.

### 2.2. Methods

All children were subjected to the following:

- medical history including symptoms related to liver disease, for example, right hypochondrial pain or swelling, jaundice, abdominal distension, general fatigue, intestinal bleeding, or itching;
- clinical assessment including general and abdominal examination;
- anthropometric measurements as follows:
  - height was measured to the nearest 1.0 mm with a Harpenden wall mounted stadiometer and weight to the nearest 0.1 kg on electronic scales together with calculation of height for age SDS [7];
  - BMI was calculated using the formula weight (in kg)/height^2^ (in meters) together with calculation of BMISDS from the age- and sex-specific reference values [8];
  - measurement of TSFT and SSFT was done using the Harpenden Skinfold Caliper together with estimation of their percentiles from the age- and sex-specific reference values [9];
- Tanner pubertal staging: for assessment of pubertal status according to the standards of Tanner and Whitehouse (1976) [10];
- Laboratory assays as follows:
  - complete blood picture;
  - liver function tests (ALT, AST, bilirubin, and albumin) assayed on Synchron CX7 Beckman Coulter Incorporation (Brea, CA, USA);
  - prothrombin time (PT);
  - serum acylated ghrelin assay after a 12-hour overnight fast using the human acylated ghrelin ELISA kit. It is an in vitro quantitative assay for detecting human acylated ghrelin peptide based on a double antibody sandwich technique.

### 2.3. Statistical Methods

The data were statistically analyzed using SPSS statistical package version 10 (Echosoft Corp., USA, 2006). Description of quantitative variables was in the form of mean ± SD, median, and range, while that of qualitative variables was given as frequency and percentage. Student's* t*-test of 2 independent samples was used to compare 2 quantitative groups for parametric data while one-way ANOVA (analysis of variance) was used to compare more than 2 quantitative groups of parametric data; when these were significant, a post hoc test was performed to compare each 2 groups separately. Pearson's correlation coefficient (*r*-test) was used to relate different variables to each other. Logistic regression analysis was used to predict risk factors. A receiver operating characteristic (ROC) curve was used to set the cutoff between cases and controls with regard to serum ghrelin levels. A value of *P* < 0.05 was considered significant.

## 3. Results

### 3.1. Cases versus Controls

All anthropometric measurements were significantly lower among cases than controls. Regarding laboratory parameters, hemoglobin and serum albumin were significantly lower while liver enzymes, total bilirubin, PT, and serum ghrelin were significantly higher among cases (Table 1).

### 3.2. Clinical and Laboratory Data according to Severity of CLD

Among studied cases, going from child A to C, there was a progressive decrease in all anthropometric parameters, hemoglobin, and serum albumin with a progressive increase in liver enzymes, total bilirubin, PT, and serum ghrelin (Table 2). On performing a post hoc test, there was a significant difference on comparing each 2 groups separately (*P* < 0.05).

### 3.3. Serum Ghrelin among Cases with CLD

**Figure 1:**
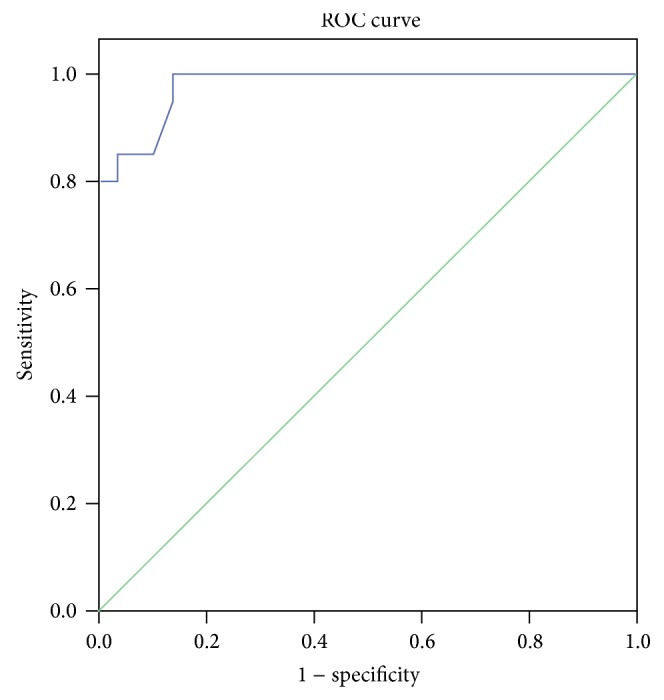
ROC curve for serum ghrelin.

Due to lack of a normal reference range for serum ghrelin in pediatric age group, a ROC curve was done to set a cutoff point for serum ghrelin between cases and controls. Accordingly the cutoff point for ghrelin between cases and controls was >65 pg/mL with a sensitivity 89.7%, specificity 95.5%, a positive predictive value (PPV) 96.3%, a negative predictive value (NPV) 83.6%, and accuracy 0.98 (Figure 1). Of the 40 studied patients, 25 (62.5%) had high serum ghrelin (>65 pg/mL). On comparing anthropometric and laboratory data among cases with high versus normal serum ghrelin, BMI, and its SDS, TSFT, and SSFT percentiles, hemoglobin and serum albumin were significantly lower while liver enzymes, total bilirubin, and PT were significantly higher among cases with high serum ghrelin levels (Table 3).

In addition, serum ghrelin correlated negatively with BMISDS (*r* = −0.95, *P* < 0.001), TSFT (*r* = −0.88, *P* < 0.001), and SSFT (*r* = 0.83, *P* < 0.001) percentiles and hemoglobin (*r* = −0.68, *P* = 0.038) and serum albumin (*r* = −0.91, *P* < 0.001) and positively with ALT (*r* = 0.88, *P* = 0.001), AST (*r* = 0.85, *P* = 0.01), total bilirubin, and PT (*r* = 0.76, *P* = 0.01).

### 3.4. Multivariate Regression Analysis to Evaluate Association between Serum Ghrelin Level and Anthropometric Parameters

Higher ghrelin was associated with lower BMISDS and TSFT and SSFT percentiles but did not affect height SDS (Table 4).

## 4. Discussion

CLD and cirrhosis are two of the most important health problems according to the current gastroenterology literature. Cirrhotic patients have nutrient and energy metabolism imbalance, which lead to malnutrition which seriously affects their prognosis [11]. Malnutrition in CLD is induced by several mechanisms, such as poor food intake, disturbances in absorption and digestion of nutritional substances in the gastrointestinal tract, and impaired hepatic synthesis of energy substrates. These abnormalities gradually affect their anthropometric parameters and cause hypoalbuminemia in those patients. Energy metabolism status in CLD is similar to that observed in accelerated starvation in healthy subjects. Reduced glucose oxidation is explained by lower production of glucose from glycogen, decreased peripheral glucose use, and decreased hepatic glycogen storage. In contrast, increased fat oxidation is considered to be due to massive lipogenesis and subsequent fat oxidation or the loss of substantial amounts of body fat mass [5].

The pathogenesis of anorexia in CLD is complex and the appetite-modulating hormone ghrelin could be involved. Acylated ghrelin is the biologically active form that modifies insulin sensitivity and body composition. Liver failure causes decreased protein synthesis and enhanced protein breakdown, which together with anorexia and reduced food intake can lead to severe protein energy malnutrition [12].

Ghrelin is a 28-amino-acid peptide produced by the oxyntic cells of the stomach. It is an endogenous ligand for the GHS-R type 1a [13]. It stimulates appetite and food intake and plays a role in meal initiation [14]. As ghrelin has a potential orexigenic effect by manipulating the melanocortin system, these neuropeptide and peptide agonists have been considered for cachexia treatment [15]. There are two major circulating forms of ghrelin: acyl and des-acyl ghrelin. Acylated ghrelin was proved to be highly relevant in the development of metabolic disturbances [16] which was the form measured in our patients. As ghrelin anticipates the initiation of meals and releases GH, it has been postulated that ghrelin integrates anabolic changes in the body as it may act as a physiological regulator of energy balance in an extensive range. In catabolic situations like in cirrhosis, raised ghrelin levels may induce a combination of enhanced food intake, increased gastric emptying, and food assimilation [15].

Our study revealed a higher serum ghrelin in patients with CLD than controls which was higher with progress of Child's grade and with deterioration of liver functions. Our findings were supported by the results of Tacke et al. (2003) [1] who reported that serum ghrelin levels in CLD patients were significantly elevated when compared with healthy controls; also, elevated levels were seen in patients having a Child's classification of grade C and severe complications (gastrointestinal bleeding, ascites, and hepatic encephalopathy). Since the likelihood of metabolic decompensation and clinical complications increases with Child's classification [17], ghrelin could potentially counteract these challenges in the Child C cirrhosis by its various metabolic functions like the modulation of energy balance, stimulation of appetite, and food intake. Similarly, El-shehaby et al. (2010) [18] found that acylated ghrelin levels showed negative correlations with serum albumin and prothrombin concentration and positive correlations with total and direct bilirubin, which indicates that the biologically active form of ghrelin is more correlated with severity of CLD. Thus, they suggested that based on the previous hypothesis, serum ghrelin decreases with improvement of liver functions and nutritional status [18]. On the other hand, other studies [5, 12] found that plasma ghrelin levels in CLD patients were not higher than in healthy controls and were not related to severity of liver damage but were closely associated with food intake in disease-associated malnutrition.

Hyperghrelinemia was evident in 62.5% of our CLD cases with negative correlations between serum ghrelin and BMISDS and TSFT and SSFT percentiles which was further supported by the association of higher ghrelin with lower BMISDS, and TSFT and SSFT percentiles proved by multivariate logistic regression analysis. In addition, BMISDS, and TSFT and SSFT percentiles were lower among patients with elevated versus nonelevated ghrelin. Thus, hyperghrelinemia was associated with a state of malnutrition in our CLD patients. Our results are concordant with those of Tacke et al. (2003) [1] and Hiroya Takahashi et al. (2006) [5] who concluded that serum ghrelin levels reflected the malnutrition state of patients with CLD being negatively correlated with anthropometric parameters, which was the case in our study. Moreover, El-shehaby et al. (2010) [18] reported that both forms of ghrelin are inversely correlated with the anthropometric parameters in patients with CLD suggesting that fasting ghrelin levels may reflect malnutrition status in CLD. They hypothesized that elevated plasma levels of both acylated and total ghrelin makes it unlikely that alterations in ghrelin secretion are directly responsible for the pathogenesis of malnutrition in CLD patients. Although ghrelin does not seem to play a direct causative role in malnutrition, increased bioactive ghrelin secretion in cirrhotic patients may reflect an adaptive mechanism signaling the hypothalamus to increase appetite and preserve energy balance in response to their poor nutritional state. Such patients with poor appetite, however, may be insensitive to the orexigenic actions of ghrelin. Desensitization of the hypothalamic ghrelin receptor, GHS-R, implicated in the control of food intake could explain in part the paradoxical response of increased ghrelin and decreased feeding leading to a malnutrition state [18]. Moreover, Marchesini et al. [19] observed the relationship of fasting ghrelin with food intake in CLD-associated malnutrition. They concluded that, in the presence of anorexia, hyperghrelinemia might indicate a compensatory mechanism trying to stimulate food intake, which is nonetheless ineffective in the physiological range [19].

In conclusion, hyperghrelinemia may represent a compensatory mechanism trying to overcome the malnutrition state complicating CLD and can be used as a parameter for early detection and assessment of the severity of malnutrition in children with CLD. Further studies to detect the role of serum ghrelin in regulation of nutritional status in children with CLD are warranted.

## Figures and Tables

**Table 1:** Clinical and laboratory data among cases and controls.

	Cases (*n* = 40)	Controls (*n* = 40)	*t*	*P*
Age (years)	7.55 ± 3.46 (1–16)	7.6 ± 2.50 (1–15.5)	1.24	0.22
Height SDS	−1.75 ± 1.58 (−3.82–0.95)	+0.69 ± 1.7 (0.99–1.84)	5.71	**0.03∗**
BMI (Kg/m^2^)	14.2 ± 1.06 (12.5–18.71)	20.2 ± 0.91 (14.1–23.9)	8.89	**0.0001∗∗∗**
BMI SDS	−1.85 ± 0.21 (−3.15–0.98)	+0.89 ± 0.28 (0–1.54)	6.11	**0.0001∗∗∗**
TSFT percentile	16 ± 5.5 (4.15–55.70)	66 ± 6.5 (25.15–78.70)	21.80	**0.0001∗∗∗**
SSFT percentile	18 ± 5.5 (6.15–57.70)	65 ± 4.4 (23–77.70)	19.89	**0.0001∗∗∗**
Haemoglobin (g/dL)	10.4 ± 1.19 (9–12.5)	13 ± 1.33 (10.5–14.5)	7.11	**0.01∗**
ALT (IU/L)	39.6 ± 13.35 (18–98)	15.65 ± 3.35 (19.5–34.0)	7.22	**0.01∗**
AST (IU/L)	36 ± 10.25 (19–70)	12 ± 2.5 (5.12–32.5)	6.55	**0.01∗**
Total bilirubin (mg/dL)	1.94 ± 0.88 (0.5–5.89)	0.5 ± 0.1 (0.2–1.0)	5.66	**0.03∗**
Albumin (g/dL)	3.05 ± 0.46 (1.89–4.2)	4.88 ± 0.56 (3.82–5.92)	4.89	**0.04∗**
PT (seconds)	14.42 ± 1.64 (12–18.7)	11.5 ± 1.20 (10–12.45)	5.10	**0.03∗**
Ghrelin (pg/mL)	130 ± 46.13 (40–210)	38.4 ± 18.11 (12–62)	15.98	**0.0001∗∗∗**

Results are expressed as mean ± SD and range; ^*^ *P* < 0.05 and ^***^ *P* < 0.001, SDS: standard deviation score, BMI: body mass index, TSFT: triceps skin fold thickness, SSFT: subscapular skin fold thickness, ALT: alanine aminotransferase, AST: aspartate aminotransferase, and PT: prothrombin time.

**Table 2:** Clinical and laboratory data according to severity of liver disease among studied cases (*n* = 40).

	Child A (*N* = 15)	Child B (*N* = 15)	Child C (*N* = 10)	*F*	*P*
Age (years)	6.5 ± 4.5 (1–13.5)	5.2 ± 6.2 (0.5–15)	7.6 ± 3.0 (1–16)	0.19	0.75
Height SDS	−1.75 ± 1.6 (−2.06–0.95)	−2.28 ± 0.46 (−3.02–0.12)	−3.36 ± 0.51 (−3.82–0.33)	6.81	**0.04∗**
BMI (Kg/m^2^)	16.10 ± 0.51 (15–18.71)	14.10 ± 0.51 (13.7–18)	13.19 ± 0.12 (12.5–17.5)	7.22	**0.031∗**
BMI SDS	−1.12 ± 0.62 (−1.4–0.98)	−1.5 ± 0.66 (−2.43–0.34)	−2.1 ± 0.72 (−3.15–0.88)	6.89	**0.030∗**
TSFT percentile	53 ± 1.5 (15–55.7)	25 ± 5.5 (18–50.5)	12 ± 4.5 (4.15–48)	8.77	**0.02∗**
SSFT percentile	54 ± 4.4 (20–57.7)	28 ± 2.3 (20–50.4)	15 ± 4.7 (6.15–45)	6.88	**0.021∗**
Haemoglobin (g/dL)	11.1 ± 1.19 (9.5–12.5)	12.3 ± 0.25 (10–12.5)	9.8 ± 0.99 (9–12.4)	5.56	**0.04∗**
ALT (IU/L)	38 ± 12.35 (18–66)	55 ± 10.15 (20–85)	82 ± 11.30 (58–98)	9.33	**0.030∗**
AST (IU/L)	29 ± 5.15 (19–54)	42 ± 10.05 (22–65)	58 ± 8.25 (28–70)	6.18	**0.04∗**
Total bilirubin (mg/dL)	0.8 ± 0.28 (0.55–3.1)	1 ± 0.11 (0.5–3.8)	1.5 ± 0.08 (0.6–5.89)	5.91	**0.04∗**
Albumin (g/dL)	3.30 ± 0.30 (2.9–4.2)	2.42 ± 0.17 (2.20–2.60)	2.01 ± 0.11 (1.89–2.99)	5.62	**0.032∗**
PT (seconds)	12.5 ± 0.24 (12–14.80)	14 ± 1.62 (12.5–17.8)	16 ± 1.20 (12.5–18.7)	6.01	**0.041∗**
Ghrelin (pg/mL)	99 ± 16.03 (40–125)	132 ± 19.23 (57–173)	189 ± 12.5 (60–210)	28.19	0.0001∗∗∗

Results are expressed as mean ± SD and range; ^*^ *P* < 0.05, and ^***^ *P* < 0.001; SDS: standard deviation score, BMI: body mass index, TSFT: triceps skin fold thickness, SSFT: subscapular skin fold thickness, ALT: alanine aminotransferase, AST: aspartate aminotransferase, and PT: prothrombin time.

**Table 3:** Clinical and laboratory data among cases with normal versus elevated serum ghrelin.

	Elevated ghrelin (*n* = 25)	Nonelevated ghrelin (*n* = 15)	*t*	*P*
Age (years)	7.50 ± 3.46 (1–16)	7.6 ± 1.50 (2–15.5)	0.24	0.52
Height SDS	−1.85 ± 1.58 (−3.11–0.95)	−1.70 ± 1.4 (−3.82–0.95)	0.71	0.31
BMI (Kg/m^2^)	13.2 ± 0.56 (12.5–15.71)	16.89 ± 0.20 (13.5–18.71)	7.89	**0.001∗∗**
BMI SDS	−2.25 ± 0.31 (−3.15–0.88)	−1.05 ± 0.11 (−2.12–0.98)	5.91	**0.01∗**
TSFT percentile	12 ± 4.5 (4.15–45.6)	36 ± 6.7 (22–55.70)	19.80	**0.0001∗∗∗**
SSFT percentile	14 ± 3.5 (6.15–47.50)	40 ± 5.5 (15.8–57.70)	17.89	**0.0001∗∗∗**
Haemoglobin (g/dL)	10 ± 0.99 (9–11.8)	11.8 ± 0.20 (9–12.5)	4.33	**0.04∗**
ALT (IU/L)	52.6 ± 12.35 (22–98)	28 ± 10.05 (18–65)	6.12	**0.02∗**
AST (IU/L)	54 ± 7.25 (28–70)	24 ± 5.15 (19–55)	8.55	**0.01∗**
Total bilirubin (mg/dL)	3.04 ± 0.78 (0.9–5.89)	0.98 ± 0.24 (0.5–2.3)	6.66	**0.02∗**
Albumin (g/dL)	2.05 ± 0.26 (1.89–4.0)	3.61 ± 0.16 (2.1–4.20)	4.23	**0.041∗**
PT (seconds)	16.02 ± 1.34 (12.5–18.7)	13 ± 1.23 (12–17.6)	6.10	**0.03∗**
Ghrelin (pg/mL)	170 ± 16.13 (72–210)	51 ± 10.12 (40–62.5)	18.91	**0.0001∗∗∗**

Results are expressed as mean ± SD and range; ^*^ *P* < 0.05, ^**^ *P* < 0.01, and ^***^ *P* < 0.001; SDS: standard deviation score, BMI: body mass index, TSFT: triceps skin fold thickness, SSFT: subscapular skin fold thickness, ALT: alanine aminotransferase, AST: aspartate aminotransferase, and PT: prothrombin time.

**Table 4:** Multivariate regression analysis to evaluate association of serum ghrelin with low anthropometric parameters among studied cases (*n* = 40).

	Odds ratio	95% CI	*P*
Height SDS	0.3	0.05–2.98	0.54
BMI SDS	5.6	1.47–6.12	<**0.01∗∗**
TSFT percentile	6.5	2.57–9.10	<**0.001∗∗∗**
SSFT percentile	4.23	1.5–3.87	<**0.001∗∗∗**

^**^ *P* < 0.01 and ^***^ *P* < 0.001; SDS: standard deviation score, TSFT: triceps skin fold thickness, and SSFT: subscapular skin fold thickness.
